# Seeing Affective and Psychotic Disorders through Addictions: Case Studies to Review the Challenges for the Survival of both “the Patient on the Edge” and “Therapeutic Alliance”

**DOI:** 10.1192/j.eurpsy.2025.199

**Published:** 2025-08-26

**Authors:** B. D. Ulug

**Affiliations:** Psychiatry, Hacettepe University School of Medicine, Ankara, Türkiye

## Abstract

**Abstract:**

Comorbidities in addiction: It is a rule rather than an exception. The story starts in childhood; even before, in infancy, and may be in prenatal life. The dimensional traits have already been there, existing obviously far before any DSM-5 diagnosis. Among these traits, the developmental features named as stress sensitivity, impulsivity and emotion dysregulation are the leading ones. Comorbidity research addressed childhood abuse, neglect or other childhood adverse experiences as a definite risk factor for adolescence and adult mental disorders, particularly substance use disorders. Developmental and environmental adversities in a mutually amplifying pattern make a vicious cycle in which the individual finally finds an illusionary exit, a pathway to addiction.

This presentation aims to discuss the complexities and challenges for the diagnosis and treatment of cases presenting with ASUD (alcohol and substance use disorders) and comorbid neurodevelopmental and affective or psychotic disorders . The history as well as the life and the management charts of the patients are reviewed in the light of information collected during the follow-up years revealing significant alterations with regard to diagnoses and therapeutic approaches. A specific focus of the case studies will be the missed or mis-diagnoses, and the impact of them on the treatment courses and the outcomes. One of the case studies with an eight year follow-up period, shows ADHD traits, alcohol use disorder, affective disorder and a later emerging severe stimulant use disorder. The second case with a similar ADHD history, presents with a stimulant use disorder, co-occuring with a severe psychotic disorder, that has been mis-diagnosed as schizophrenia. The life and management charts of the studied cases convey the drawbacks of the diagnostic difficulties, the treatment failures and the implication of efficient therapeutic strategies.

The challenges faced by clinicians due to co-occuring disorders that have become a common practice for addiction professionals. should be managed by transdiagnostic and integrative modalities. While big data or empirical large datasets can have their own limitations to help the practitioner for overcoming such complexities of real world situations, as stated in Stein’s article (2022, Psychiatric diagnosis and treatment in the 21st century: paradigm

shifts versus incremental integration) “the age-old single-case studies, may sometimes provide clinical insights that outweigh those from big data analyses.”

**Disclosure of Interest:**

None Declared

